# Oral malignant melanoma of the lower jaw—a case report with immunohistochemical investigations

**DOI:** 10.3332/ecancer.2023.1561

**Published:** 2023-06-15

**Authors:** Sandhya Tamgadge, Treville Pereira, Amit Date, Priyadarshini Kalimuthu, Avinash Tamgadge

**Affiliations:** 1 Department of Oral and Maxillofacial Pathology and Microbiology, School of Dentistry, D. Y. Patil University, Sector 7, Nerul, Navi Mumbai 400706, Maharashtra, India; 2 Department of Oral and Maxillofacial Surgery, School of Dentistry, D. Y. Patil University, Sector 7, Nerul, Navi Mumbai 400706, Maharashtra, India

**Keywords:** oral manifestations, oral mucosal melanoma, malignant

## Abstract

Oral mucosal melanoma is a type of pigment-producing cell malignancy that primarily affects the skin and oral mucosa, but can also affect the ears, eyes, gastrointestinal tract and vaginal mucosa. Oral mucosal melanoma has several different clinical manifestations. Even though it frequently manifests as a black-brown patch, macule, or nodular lesion with varying tones of red, purple or depigmented tissue, the clinical characteristics and pathobiological behaviour of oral mucosal melanomas differ from those of cutaneous melanomas. The prognosis for oral melanomas is exceedingly bad because they frequently exhibit no symptoms, which may delay diagnosis. The case of a 65-year-old male patient with a primary complaint of blackened gums in the right lower back region of the jaw is presented here.

## Introduction

Weber published the first description of oral mucosal melanoma in 1859 [1]. The oral mucous membrane’s basal layer contains melanocytes that can grow out of control, leading to the extremely rare tumour known as malignant melanoma of the oral cavity. Melanocytes are generated from the neural crest and migrate to the skin, mucous membranes and various other locations [2]. Over the past several decades, there has been a higher incidence of melanoma worldwide [1, 3]. The cutaneous and ocular forms of melanoma are the most prevalent types [4]. Only 0.5% of all head and neck neoplasms are mucosal melanomas, which is rare and affects the sinonasal cavity, oral cavity, pharynx, larynx and upper oesophagus [4, 5].

Eighty per cent of oral lesions have been prevalent in the maxillary gingiva, hard palate and alveolar ridge and 20% of cases have been in the mandible [4].

There is a male predominance, with a ratio of about 2:1 between males and females. Patients under the age of 20 are extremely uncommon to get oral mucosal melanoma, which primarily affects people over the age of 40. Males between 51 and 60 years are more likely to have oral malignant melanoma than females with the age range between 61 and 70 years [6]. Mucosal melanomas have no recognised risk factors, unlike cutaneous melanomas, that are etiologically in relation to sun exposure [4, 2, 6].

The oral mucosa is constantly exposed to chemical, thermal and physical events, such as alcohol consumption, smoking, poor oral hygiene and irritation from teeth, dentures and other oral appliances (crown and bridge), yet there is no clear connection between melanomas and these factors. Although often benign, intraoral melanocytic proliferations (nevi) may exist and may be the cause of some oral melanomas; nevertheless, the exact course of events in the oral cavity is largely unexplored. Today, it is believed that the majority of oral melanomas develop spontaneously [7].

Despite being extremely rare, the malignant transformation of nevi into melanoma entails the clonal development of cells that attain a selective growth advantage. A nevus that already exists or a single melanocyte in the basal cell layer must undergo this change before the altered cells can multiply in any dimension. Oral melanomas typically have an asymmetrical shape, dark brown to a black colour and exist without any symptoms. Significant swelling, tooth mobility, ulceration, or haemorrhages of the underlying epithelium are frequent signs of the lesion [7].

However, a small percentage of oral mucosal melanoma cases include clinical manifestations such as painless, white, mucosa-coloured or red masses, which can lead to a delayed diagnosis, treatment delay and a worse probability of survival. The non-pigmented mixed type is an amelanotic nodular tumour surrounded by a radial phase of growth, as opposed to the non-pigmented nodular type, which lacks a radial phase of growth. The biological activity of the majority of oral melanomas is aggressive, and they frequently metastasize, recur and die. However, compared to pigmented melanoma, amelanotic melanomas have a worse prognosis [8, 9]. Here, we have described a rare instance of mandibular malignant melanoma.

## Case report

A 65-year-old male patient reported to the outpatient department with a primary complaint of blackening of gums in the right lower back region of the jaw for about 6 months. The swelling was initially small and it gradually reached its current size. The patient was a chronic smoker, consuming about 10–15 cigarettes per day for 30 years. There was no history of any underlying conditions or head and neck injuries.

**Figure 1.**
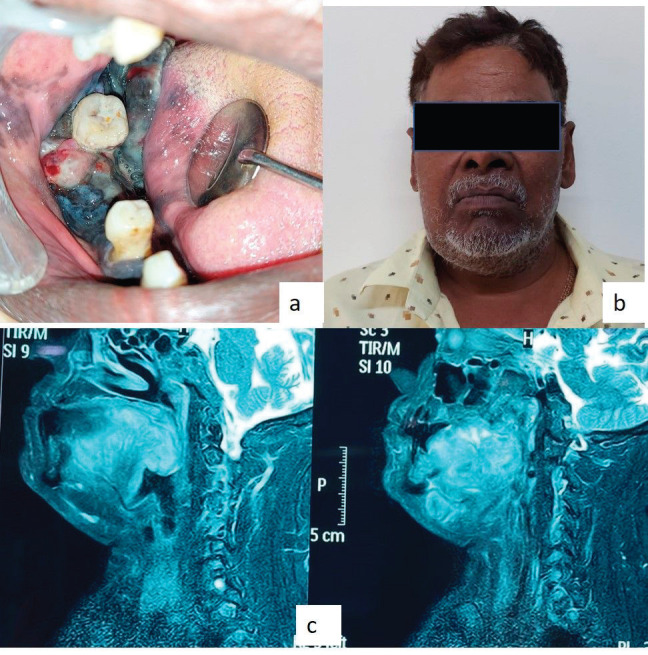
(a–d): Magnetic resonance imaging (MRI) image shows the T2W sagittal section of the lesions.

On extraoral examination, right submandibular lymph node was tender, sensitive and firm on palpation. An intraoral clinical examination revealed a diffuse, expansile, non-tender, pigmented (bluish black in colour) growth that exhibited a sessile, irregular, nodular and ulcerative surface. The involved teeth were appeared to be completely surrounded by the lesions. The dimension was approximately 5.5× 4.5× 3.0 cm in size covering the entire right mandibular alveolar ridge of the right mandibular canine region anteriorly and posteriorly extended till the retromolar region. The lesion involved the entire lateral surface of the tongue, and crossing the midline of the soft palate. Superioinferiorly the lesion was extending from the marginal gingiva (in relation to 43, 45, 46 regions) to the floor of the mouth (Figure 1).

The complete blood cell count was unremarkable and was within normal ranges. Urine analysis to detect melanin metabolite was done, and it showed insignificant results [10].

**Figure 2.**
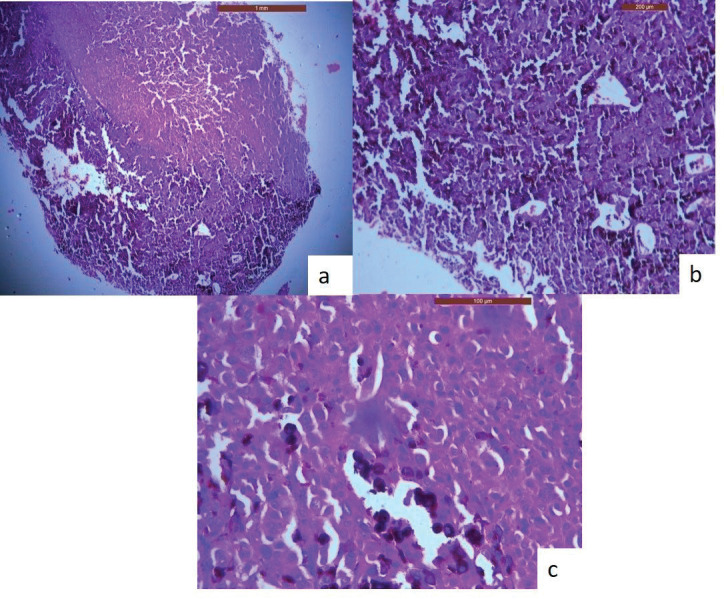
H & E stained sections of oral malignant melanoma. (a): A-4× view of the lesion shows the lesional tissue which is composed of malignant melanocytes infiltrating into the connective tissue stroma. (b): B-10× melanocytes are infiltrating into the connective tissue stroma in the form of sheets, chords. (c): C-40× melanocytes are round in shape, exhibit nuclei of varying sizes which occupies nearly the entire cell cytoplasm.

**Figure 3.**
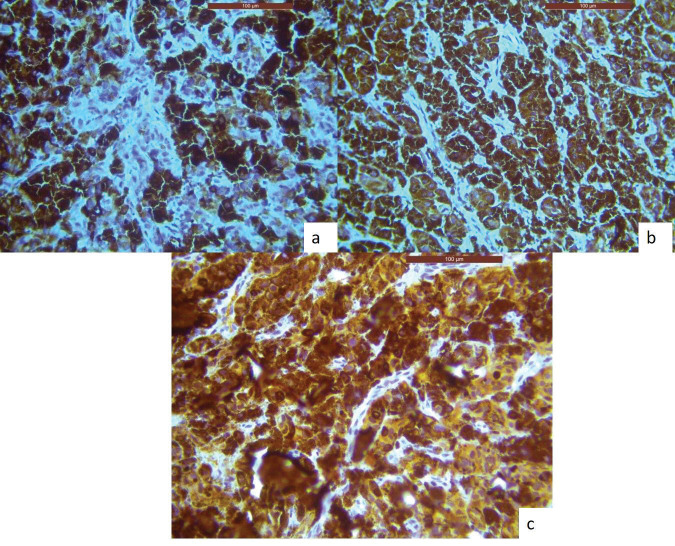
IHC analysis reveals lesional tissue is positive for (a): HMB-45, (b): Melan-A and (c): S-100 markers, respectively.

An incisional biopsy was done, and the histological analysis revealed that the lesional tissue was predominantly composed of melanocytes in the form of sheets, chords infiltrating into the underlying connective tissue stroma. Then invading melanocytes were round in shape, exhibited nuclei of varying sizes occupying nearly the entire cell cytoplasm. Tumour cells contained varying amounts of melanin deposition. The connective tissue stroma showed dysplastic characteristics indicative of malignant melanoma, such as cellular atypia and anisocytosis (Figure 2a–c). Malignant melanoma can be easily diagnosed using routine histology but immunohistochemical stains can be used to evaluate prognosis. Thus, antibodies to S-100, homatropine methylbromide 45 (human melanoma black (HMB45)), and melan A showed strong positive for tumour cells (Figure 3a–c).

**Figure 4.**
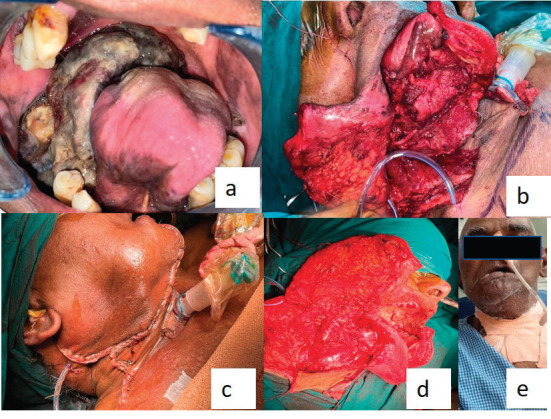
(a): Post-operative image of the second follow-up (after the incisional biopsy) represents that the lesion was aggravated and completely obliterating the pharyngeal spaces. (b–d): Operative images. (e): Extraoral postoperative.

The final diagnosis of malignant melanoma was considered on the account of clinical, radiological and histopathological findings. Intraoral examination revealed a highly aggravated lesion after 2 weeks after the first incisional biopsy. Immediate surgery was planned as it had obscured the oropharyngeal spaces (Figure 4).

The sagittal section of the face **T2 weighted image (**T2W**)** MRI (images showed, a well-defined isointense lesion involving the musculature of the posterior third of the tongue. The lesion obliterated the oropharynx and involved the posterior wall of the pharynx. The overall findings were suggestive of malignant lesions (Figure 1). The patient did not go for a positron emission tomography scan as he was from a low socioeconomic background.

The diagnosis, staging, available treatment protocols and prognosis of the diseases were explained to the patient and his family members. The patient was directed to a medical hospital at the institute. A tracheostomy was performed under general anaesthesia. Skin, subcutaneous tissue platysma layer was incised after giving right-sided Crile’s and *Schobinger’s incision*. Necrotic positive lymph nodes 1B and IIA were noted. IA lymph nodes were excised. Contra lateral anterior belly of digastric and sternocleidomastoid was demarcated. A spinal accessory nerve at the level of IIA was identified. Nodes with respect to II A, IIB and IIIA near the internal jugular vein and spinal accessory nerve were identified. Dissection was done with IIIA, IIIB, IVA, IVB and V, sternocleidomastoid, spinal accessory and internal jugular vein.

The neck incision extended over the lower lip as the midlines split. Control was taken over the lingual artery above the superior thyroid.

Primary tumour resection was done with the lateral border over buccal mucosa, 1 cm above the right lower gingivo buccal sulcus, medially along the symphysis, the floor of the mouth on the right lateral aspect of lingual frenum while including the lateral border of the tongue and tongue muscles at the junction of anterior one third and posterior two-thirds of the tongue.

The posterior border at vallecula, posterolateral border including tonsillar fossa and hemimandible, anteromedial hyperpigmented area on alveolar mucosa was sent for frozen section to ensure negative margin. A verbal report suggestive of a negative margin was received. Primary closure was done on the tongue. Haemostasis was achieved. A drain was secured and a parotid stitch was given. Control over the lingual artery was released. All teeth were extracted, haemostasis was achieved. The patient was handed over to the plastic surgeon for pectoralis major myocutaneous flap (PMMC). Neck closure was done.

A second follow-up was done after 2 weeks, for the healing of surgical wounds. Unfortunately, the patient passed away 2 months after surgery (Figure 4). American Joint Committee on Cancer (AJCC) staging was PT4aN1 at the time of surgical excision and the worst pattern of invasion was four.

## Discussion

Oral mucosal melanomas are considered highly aggressive neoplasms with high mortality rates. Melanoma of the head and neck account for around 25% of all melanomas, but mucosal melanomas are rare, accounting for less than 1% of all melanomas [11]. Any minor surgical procedures might lead to invasiveness which was observed in our case after an incisional biopsy. 80% of melanomas are usually seen in the maxilla but our case was seen in the mandibular gingiva which could be considered a rare finding [12]. It has been stated that adipose tissue in submucosa triggers the aggressiveness of melanocytes when melanocytes shifts from radial to vertical growth phase [13].

Cutaneous melanocytes are known for protecting from Ultraviolet (UV) radiation but oral melanocytes are known for their role in the inflammatory process rather than UV protection. The oral cavity is always under constant mechanical, microbial and chemical stress which might stimulate oral melanocytes to become pathologically altered. In several situations where there is chronic local irritation to the oral mucosa, for example in cheek biting, oral lichen planus, tobacco smoking and non-amalgam dental restorations as well as in some leukoplakias, the oral mucosa shows a lot of melanin pigmentation as stated by Tolleson [14] and Barrett and Scully [15]. In our case, too some chronic irritation factor could have been present as the patient was a tobacco chewer.

The physical characteristics and biological behaviour of ORAL MUCOSAL MELANOMAS (OMM) may exhibit great heterogeneity, which may make the clinical diagnosis very challenging [12, 5]. Amalgam tattoo, Nevi, post-inflammatory pigmentation, melanotic macule, melanoacanthoma, smoking-associated melanosis, melanoplakia, Addison's illness, Peutz-Jeghur syndrome and Kaposi's sarcoma are all included in the differential diagnosis [4, 12]. Intraoral mucosal melanoma most of the time go unnoticed in the initial phases as it might be mistaken for gingival pigmentations, especially in Indian patients with low socioeconomic background.

A comprehensive review of oral mucosal melanomas has been reported by Vikey and Vikey [12], Barrett and Scully [15] and Tolleson [14].

The majority of researchers have found that oral mucosal melanoma are extremely aggressive tumours, and a number of variables, such as late detection, poor resectability and early metastasis, contribute to their invasion. Age, the size of the oral original tumour, the difficulty of resecting it, non-pigmented (amelanotic) lesions and the involvement of local lymph nodes all directly correlate with a worse prognosis. Additionally, abnormal melanocytes high mitotic rate and vascular or brain invasion are other variables linked to a bad prognosis. In our case, the patient was in stage I at the time of the diagnosis, according to Munde *et al* [17] and Sahana Ashok *et al* [18]. Patients with low socioeconomic backgrounds usually have poor prognoses due to limited financial resources, especially in India which was seen in our case. The patient survived only for 2 months after complete resection due to the sudden spread of the lesion.

The clinical diagnosis, in this case, was supported by the tumour cells' positivity for HMB-45, S-100 protein and Melan-A or MART-1. Grading was considered as per the eighth edition of AJCC [19, 20].

Broad surgical excision with appropriate negative margins, neck dissection with or without radiation and chemotherapy are the usual therapies for malignant melanoma. In contrast to cutaneous melanoma, OMM may be treated with radiotherapy if the condition is localised [5].

The five-year survival rate has been reported in the literature but the current case died within 2 months of excisional biopsy [21]. Melanoma is known for aggressive behaviour. The current case showed the same behaviour after incisional biopsy and spread aggressively to involve the oropharynx [22].

Chemotherapy or immunotherapy may be used as adjuvant to prevent recurrence [12].

## New information

1. A case of oral melanoma showing sudden aggravation in just 1 month after incisional biopsy.
2. A very advanced case of oral mucosal melanoma has been reported. There is nothing new about the findings, but it showcases the advanced presentation in Low- or Middle-Income Countrys. This population has financial limitations as far as investigations and regular follow-up are concerned.
3. Melanoma is usually found in the maxilla but our case was seen in the mandibular gingiva.

## Conclusion

It is necessary to report such cases because of their distinctive morphological characteristics, extremely low frequency and aggressive behaviour. A high index of suspicion allows for an earlier diagnosis and improved prognosis.

## Consent

Patient’s informed consent was taken for the documentation.

## Financial support and sponsorship

Nil.

## Conflicts of interest

The authors declared they have no competing interests.
